# A Culture Change: Impact of a Pediatric Antimicrobial Stewardship Program Based on Guideline Implementation and Prospective Audit with Feedback

**DOI:** 10.3390/antibiotics10111307

**Published:** 2021-10-27

**Authors:** Bindiya Bagga, Jeremy S. Stultz, Sandra Arnold, Kelley R. Lee

**Affiliations:** 1 Department of Pediatrics, Division of Pediatric Infectious Diseases, University of Tennessee Health Science Center, Memphis, TN 38163, USA; sarnold5@uthsc.edu; 2 Le Bonheur Children’s Hospital, Memphis, TN 38103, USA; Kelley.Lee@lebonheur.org; 3 Department of Clinical Pharmacy and Translational Science, University of Tennessee Health Sciences Center, Memphis, TN 38163, USA

**Keywords:** antimicrobial stewardship, pediatric, antimicrobial resistance, antibiotics, sepsis

## Abstract

Reports analyzing the impact of pediatric antimicrobial stewardship programs (ASP) over long periods of time are lacking. We thus report our ASP experience in a pediatric tertiary referral center over a long-term period from 2011 to 2018. Our ASP was implemented in 2011. The program was based primarily on guideline development with key stakeholders, engaging and educating providers, followed by prospective audit with feedback (PAF). Monitored antibiotics included meropenem, piperacillin–tazobactam, and cefepime, followed by the addition of ceftriaxone, ceftazidime, cefotaxime, ciprofloxacin, levofloxacin, linezolid, and vancomycin at various time points. Specifically, the program did not implemented the core strategy of formulary restriction with prior authorization. Process- and outcome-related ASP measures were analyzed. We saw a 32% decrease in overall antibiotic utilization, a 51% decrease in the utilization of antibiotics undergoing PAF, and a 72% reduction in the use of broad-spectrum antibiotics such as meropenem. There was a concomitant increase in organism susceptibility and a reduction in yearly drug purchasing costs of over USD 560,000 from baseline without changes in sepsis-related mortality. Our study highlights that a pediatric ASP based primarily on the principles of guideline development and PAF can improve antibiotic utilization and institutional bacterial susceptibilities without a detrimental impact on patient outcomes by changing the culture of antimicrobial utilization within the institution.

## 1. Introduction

Antibiotics are amongst the most prescribed medications to hospitalized children, with a large majority of the use thought to be inappropriate and unnecessary [1,2]. Antibiotic misuse can lead to adverse events, increase health care costs, and contribute to the growing antibiotic resistance [3,4]. Both national and international health organizations recognize antimicrobial resistance as a threat on a global scale [5,6,7]. In response to the threat of the overuse of antibiotics, the Infectious Diseases Society of America (IDSA) and the Pediatric Infectious Diseases Society (PIDS) published evidence-based guidelines for the implementation of antimicrobial stewardship interventions [8,9,10]. These core interventions include formulary restriction with preauthorization and/or PAF. The long-term process and outcome measures of pediatric ASP are not well described. 

We implemented a pediatric ASP in a free-standing tertiary care children’s hospital in 2011. In this study, we describe the process and core strategies applied and the outcome measures since the initiation of the program. We hypothesized that education, guideline implementation, and PAF would lead to a sustained long-term improvement in our institution. To the best of our knowledge, we report the longest duration of sustained impact of a pediatric ASP to date.

## 2. Results

### 2.1. Process-Related Measures

**Figure 1:**
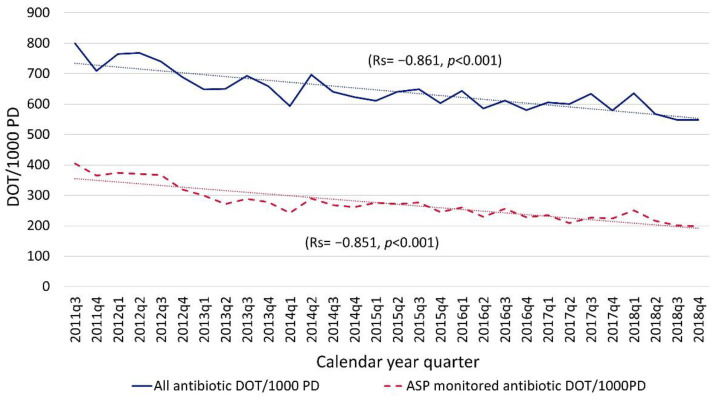
Quarterly antibiotic utilization for all antibiotics and antibiotics undergoing monitoring via PAF. Monitored antibiotics included meropenem, piperacillin–tazobactam, and cefepime, starting in 2013. Ceftriaxone, ceftazidime, cefotaxime, ciprofloxacin, levofloxacin, linezolid, and vancomycin were added at various time points after 2015. Spearman’s correlation (Rs) assessed trends over time. ASP, antimicrobial stewardship program; DOT, days of therapy; PD, patient days.

Institutional overall antimicrobial utilization decreased by 32% and the utilization of antibiotics undergoing targeted PAF by ASP team members decreased by 51% from program inception through 2018 (Figure 1). The absolute reduction in overall utilization from 2011 to 2018 was 252 DOT/1000 PD. The absolute reduction in monitored antibiotics was 206 DOT/1000PD.

**Figure 2:**
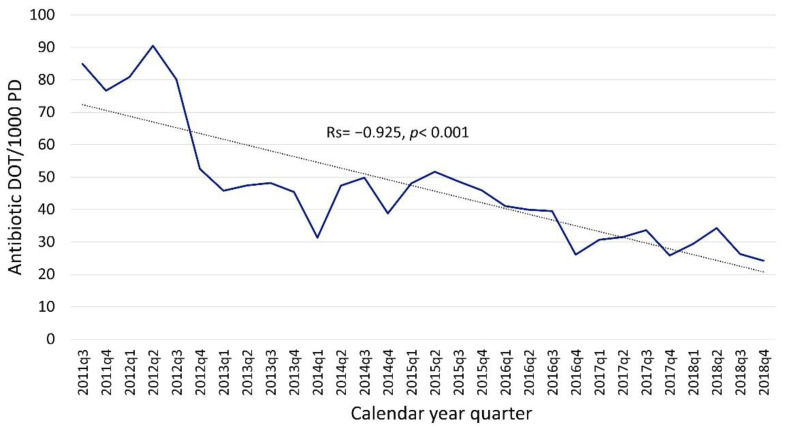
Quarterly broad-spectrum antibiotic utilization (meropenem, cefepime, piperacillin/tazobactam). These agents were monitored via PAF starting in 2013. Spearman’s correlation (Rs) assessed trends over time. DOT, days of therapy; PD, patient days.

Quarterly broad-spectrum antibiotic (e.g., meropenem, cefepime, piperacillin/tazobactam) utilization decreased by 72% from ASP initiation through 2018 with an absolute reduction of 60 DOT/1000 PD. Although broad-spectrum antibiotics were primarily targeted by our ASP and had greater relative reduction, the absolute reduction in overall antibiotic utilization seen in Figure 1 (~200 DOT/1000 PD) was greater than the absolute reduction seen in the broad-spectrum monitored antibiotics (~60 DOT/1000 PD) illustrated in Figure 2. There was also a 46% decrease in PAF-guided interventions on broad-spectrum antibiotics, from 1.4 interventions/1000 PD in 2013 when PAF was started to 0.7 interventions/1000 PD in 2018 (R = −0.959, *p* = 0.002). This suggests some of the reduction in utilization was driven by prescribers within the institution in addition to those directly promoted by the ASP program.

### 2.2. Outcome-Related Measures

**Figure 3:**
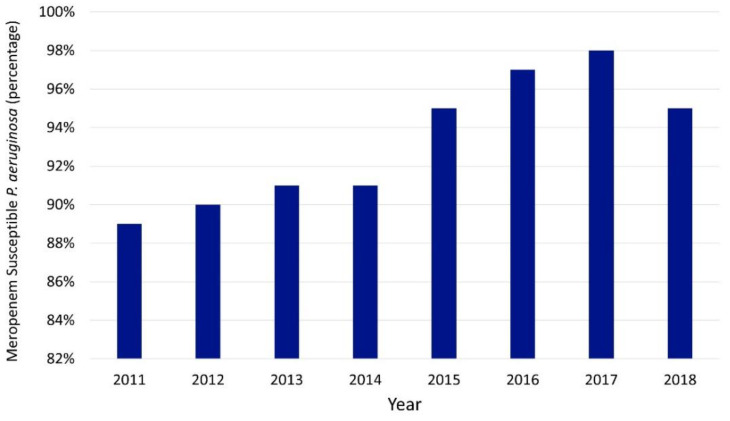
Meropenem susceptibility over time. Susceptibility changes were significant between years (*p* = 0.001 via chi-squared).

Figure 3 illustrates an increase in *P. aeruginosa* susceptibility to meropenem over time at our institution. This includes one additional year compared to our previous publication [11]. Our program has also previously reported the increased susceptibility of hospital-associated pathogens to third-generation cephalosporins with decreasing use of these drugs [12].

**Figure 4:**
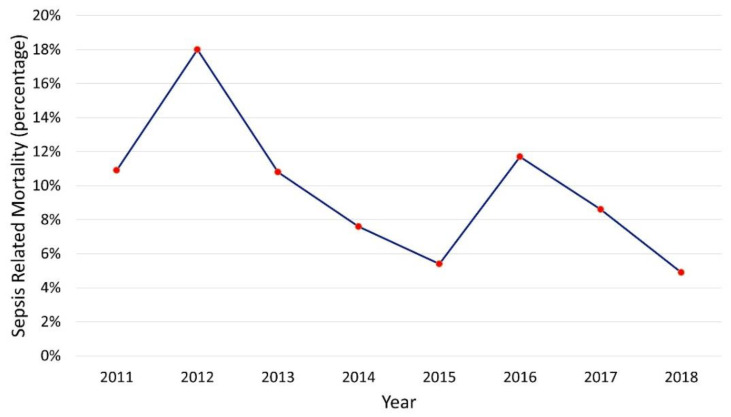
Sepsis mortality over time. Mortality attributed to sepsis did not change over time (R = −0.616, *p* = 0.104).

Sepsis-related mortality has not significantly changed at our institution since the creation of the ASP, illustrating that there were not significant adverse consequences to utilizing narrower-spectrum antibiotics (Figure 4).

**Figure 5:**
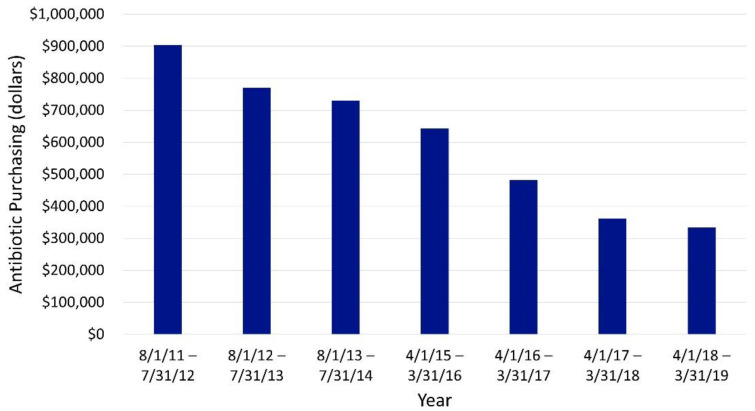
Yearly pharmacy antibiotic purchasing. Data are unavailable from 1 August 2014–31 March 2015 due to a change in wholesaler occurring during this time period.

Antimicrobial purchasing costs also decreased on a yearly basis, with a decrease of over USD 569,000 in yearly spending from initiation of the ASP through the first quarter of 2019 (Figure 5). The changes consistently decreased both before and after wholesaler change in 2014–2015. 

## 3. Materials and Methods

### 3.1. Description of ASP

An ASP was started in June 2011 at Le Bonheur Children’s Hospital, a large tertiary referral hospital in Memphis, TN with a 1 FTE pediatric pharmacist and a 0.5 FTE pediatric ID physician (position shared by two people). Another 0.5 FTE pharmacist was added in 2014. The ASP initially focused on the development of empiric antibiotic use guidelines for critical care units as those were the sites of most broad-spectrum antimicrobial use. The ASP’s guidelines for the initiation of empiric antimicrobials in critical care units were implemented throughout the 2012 calendar year as previously described [13]. In 2013, PAF of select broad-spectrum antibiotics and positive sterile-site cultures in individual patients was implemented on weekdays. When applicable, the ASP made patient-specific recommendations to the primary team, which included antimicrobial regimen changes (choice, duration of therapy, route of therapy) or infectious diseases consult as deemed applicable. All cases were initially reviewed by the ASP pharmacist who then discussed recommendations with the ASP physician. Recommendations were made to the primary team resident both verbally and electronically. A feedback loop was utilized where antibiotic utilization trends and guideline compliance were assessed and shared with relevant unit-specific physician champions and physician teams. Routine education of new providers was carried out as deemed necessary using various platforms, including pediatric grand rounds, resident conferences, and physician on-boarding sessions.

Guideline development followed by PAF for targeted antibiotics was the cornerstone strategy of initiatives for our ASP. We previously reported on guideline creation for linezolid use and urinary tract infection treatment [14,15]. Table 1 provides a complete list of ASP initiatives and guideline revisions since program initiation. Restrictions, prior authorizations, and ASP team rounding with patient care teams (handshake PAF) were not used, to maintain prescriber autonomy and promote a culture of antimicrobial stewardship driven by all prescribers within the hospital. Antifungal and antiviral stewardship have not been initiated at our institution. 

### 3.2. Statistical Analysis

This was a retrospective analysis of process and outcome measures related to the ASP efforts at our institution from October 2011 through 31 December 2018. The main process measure was institutional antibiotic days of therapy (DOT)/1000 patient days (PD) for all antibiotics and antibiotics monitored by ASP via PAF. Our outcome measures included institutional *P. aeruginosa* susceptibilities (excluding cystic fibrosis patients), sepsis-related mortality, and institutional pharmacy antibiotic purchasing costs. These measures were selected from recommended measures for tracking an ASP in the pediatric inpatient setting [9,10].

Antibiotic utilization data were collected as antibiotic DOT/1000 PD for targeted antibiotics and all antibiotics within our institution. Mortality was reported as the percentage of sepsis deaths. Utilization and mortality data were obtained from the Pediatric Health Information System Database for our institution. Pearson and Spearman’s correlations were used to assess quarterly or yearly trends from the 3rd quarter of 2011 to the 4th quarter of 2018, for parametric and nonparametric data, respectively. Chi-squared test compared susceptibilities among years. Significance was set at *p* < 0.05 for statistical analyses. Statistical tests were performed using the IBM Statistical Package for Social Sciences software version 27 (IBM Corp., New York, NY, USA, 2020). Antibiotic purchasing data were collected on a yearly basis for all antibiotics, and these were provided for descriptive purposes. There was a change in wholesaler at the end of 2014 and beginning of 2015, and accurate purchasing data could not be obtained during that transition. 

## 4. Discussion

Our study describes the long-term impact of an ASP in a free-standing children’s hospital. The strategy of restriction with prior authorization was not utilized at any time since the inception of the program. Instead, the approach focused on guideline development and provider education along with PAF with re-education as needed. Like our study, PAF was the most frequently used ASP core component strategy reported in a review of pediatric ASPs [16]. However, there was limited information noted on the other effective components of these programs in the pediatric setting. Our study adds to the literature by having a clear description of the multiple ASP strategies employed, which might be beneficial for other programs. Interestingly, along with the expected reduction in broad-spectrum antimicrobials which were targeted and monitored, there was also a substantial reduction in all antimicrobials, suggesting that there was indeed an institutional culture change of improved antimicrobial prescription practices. 

Although a change in prescription practices is a well-reported outcome of ASPs, safety, reduction in health care costs, and change in resistance patterns have been less frequently described in detail. In a recent systematic review of outcomes of pediatric ASPs globally, only 14.2% of the included studies (16/113) quantified cost savings related to the intervention [17]. Most studies (89.3%) in this review did not report a change in antimicrobial resistance patterns as an outcome measure, with only seven studies [13,18,19,20,21,22,23] showing an increased susceptibility of the bacteria analyzed. Our study adds to the existing literature on pediatric ASPs as we are reporting outcome measures beyond improved antimicrobial practices. 

During the same timeframe, outcome measures showed that sepsis mortality did not change, indicating that appropriate antimicrobials were being delivered and that patient outcomes in that aspect were not compromised by the change in antimicrobial prescription practices. It is well known that antibiotic overuse leads to the development of resistance and in the past, we have seen that in our institution as well [14]. However, the contrary is always harder to prove, as the results of antimicrobial prescription on local antibiograms may be delayed. We were able to show a change with *P. aeruginosa* as depicted above (Figure 3) and have previously shown that our ASP-driven reduction in third-generation cephalosporin utilization correlated with increased susceptibility among AmpC producers [11,12]. While the data are not shown, the trend in low carbapenem utilization and high susceptibilities compared to before ASP implementation has continued through 2020. Changes in resistance in *P. aeruginosa* with reduced carbapenem use have been reported elsewhere as well [18].

Given its retrospective nature, our study has certain limitations. Firstly, sepsis mortality is multifactorial and not just dependent on antimicrobials alone, thus other factors could have contributed favorably to this outcome data apart from antimicrobial prescription. Similarly, another limitation of our study is the possibility that the decreased cost we saw could be attributed to decreasing antibiotic cost over time (price reductions and generic medications) in addition to ASP interventions. Lastly, our rise in the *P. aeruginosa* susceptibility rates to carbapenems is similar to national trends of increasing susceptibilities to broad-spectrum agents [24]. This may be due to the national efforts of ASPs to decrease the use of these antibiotics. 

## 5. Conclusions

To conclude, we report our process and outcome metrics of our ASP in a free-standing children’s hospital. Our long-term reductions in antibiotic use show that a successful pediatric ASP is possible without the adoption of the core strategy of formulary restriction with prior authorization. Empowering physicians with education and guidelines followed by PAF loops can lead to a sustained culture change for antimicrobial utilization without compromising patient outcomes, as well as an improvement in resistance patterns and costs.

## Figures and Tables

**Table 1:** ASP initiatives timeline.

Initiative	Date
Start of stewardship program	June 2011
Critical care units’ empiric antibiotic use guidelines full implementation	August 2012
Community-acquired pneumonia guideline implementation	December 2012
Daily PAF on broad-spectrum antibiotics initiated	February 2013
Daily PAF on positive sterile-site cultures initiated	March 2014
Cystic fibrosis guideline implementation	September 2014
Linezolid use guideline implementation	February 2015
Linezolid added to daily PAF	April 2015
Third-generation IV cephalosporins and vancomycin added to daily PAF	May 2015
Practice change promoting narrower-spectrum antibiotics for perforated appendicitis	January 2016
Practice change promoting narrower-spectrum antibiotic use for UTI	August 2017
Revised/updated CVICU empiric antibiotic use guidelines	November 2017
Revised/updated PICU empiric antibiotic use guidelines	March 2018
Provider-driven 48 h antibiotic time-out implemented	April 2018
Revised/updated NICU empiric antibiotic use guidelines	June 2018
Fluoroquinolones added to daily PAF	October 2018

PAF, prospective audit with feedback; UTI, urinary tract infection; IV, intravenous; CVICU, cardiovascular intensive care unit; PICU, pediatric intensive care unit; NICU, neonatal intensive care unit.

## Data Availability

The data used in this project are not able to be deposited in a publicly available database, but additional details regarding data may be available upon request from the author. All relevant data are included in the manuscript.
